# A meta-analytic review of the relationship of cancer coping self-efficacy with distress and quality of life

**DOI:** 10.18632/oncotarget.15758

**Published:** 2017-02-27

**Authors:** Andrea Chirico, Fabio Lucidi, Thomas Merluzzi, Fabio Alivernini, Michelino De Laurentiis, Gerardo Botti, Antonio Giordano

**Affiliations:** ^1^ Department of Developmental and Social Psychology, University “La Sapienza” of Rome, Naples, Italy; ^2^ Department of Psychology, University of Notre Dame, Indiana, USA; ^3^ National Institute for the Evaluation of the Education System, Rome, Italy; ^4^ Dipartimento di Senologia, SC Oncologia Meduca Senologica, Istituto Nazionale Tumori IRCCS Fondazione “G. Pascale”, Naples, Italy; ^5^ Dipartimento di Patologia Diagnostica e di Laboratorio: SC di Anatomia Patologica e Citopatologia, Istituto Nazionale Tumori IRCCS Fondazione “G. Pascale”, Naples, Italy; ^6^ Sbarro Health Research Organization, Philadelphia, PA, USA; ^7^ Deparment of Medicine, Surgery and Neuroscience, University of Siena, Siena, Italy

**Keywords:** cancer, self-efficacy, coping, meta-analysis, quality of life

## Abstract

Self-efficacy for coping with cancer is a specific construct that refers to behaviors that occur in the course of dealing with a cancer diagnosis, cancer treatments, and transitioning to survivorship. One of the more widely used measures of self-efficacy for coping strategies with cancer is the Cancer Behavior Inventory. The following general questions provide a framework for this research: 1. Is self-efficacy for coping with cancer related to distress and quality of life of a cancer patient?. 2. Do self-efficacy for coping with cancer and the target psychological outcomes (i.e., distress and quality of life) change in longitudinal studies, with or without intervention? One-hundred eighty studies cited the different versions of the Cancer Behavior Inventory and 47 used the scale. Result showed an inverse relationship between self-efficacy for coping with cancer and distress, and a positive relationship between self-efficacy for coping with cancer and Quality of Life, both with a large effect size. The strong relationship of self-efficacy and outcomes, resulted of the specificity of the instrument, which targets specific coping strategies that are closely aligned with positive outcomes in adjusting to cancer. However, the results are consistent with the theory, which states that compared to those with low efficacy, highly efficacious people demonstrate less anxiety and better adjustment in stressful situations and consistent with prior results in which self-efficacy is positively related to quality of life.

## INTRODUCTION

The diagnosis, treatment, and long-term management of cancer can present individuals with a multitude of stressors at various points in that trajectory. The prevalence of psychological distress among cancer patients is higher than the general population, which increases the risk for developing clinical levels of anxiety and depression [1, 2]. In a recent study, Fatiregun et al showed that the prevalence of anxiety disorder in breast cancer patients ranges from 1% to 49% [3]. Psychosocial distress may appear early in the diagnostic process and have negative effects on compliance with treatment and subsequent quality of life [4]. Because not all patients experience distress, there may be factors that prevent distress or help dissipate distress after its onset.

Establishing an evidence base of factors that prevent or mitigate distress is critical to establishing interventions that might help those at risk based on protective factors such as coping skills and strategies.

Social Cognitive Theory [5] provides a framework for conceptualizing stress resistance or distress mitigation in that it posits that expectations about one's ability to perform certain behaviors may optimize the accomplishment of those target behaviors and lead to the attainment of desired goals and outcomes. In line with Social Cognitive Theory, Self-Efficacy Theory includes the premise that people who have greater confidence in their ability to execute courses of action, such as coping behaviors, have a higher probability of attaining goals, such as maintaining a desired state of quality of life and preventing or mitigating stress [6]. Moreover, those who have higher self-efficacy will persist longer in goal seeking presumably adapting their behavior strategies to increase the probability of goal attainment. Self-efficacy for coping with cancer is a specific construct that refers to behaviors that occur in the course of dealing with a cancer diagnosis, cancer treatments, and transitioning to survivorship. In a systematic review of the literature on interventions for cancer patients, it does appear that interventions that have a social cognitive focus are more effective in producing behavior change [7]

The next step in this process of investigating self-efficacy in the context of cancer is to focus meta-analytic reviews on specific measures of self-efficacy to determine their utility in relation to critical outcomes and refine the scales of those measures based on systematic, meta-analytic scrutiny. However, because each instrument focuses on different aspects of dealing with cancer, direct comparative analyses may not yield as useful results as the in depth analysis of each instrument separately.

Telch and Telch [8] constructed the first assessment measure of self-efficacy for coping with cancer that included six subscales. Because information about its development and initial psychometric properties are not available and the instrument was never vetted, peer reviewed, or published, one can assume that the authors developed the measure in conjunction with their group intervention and that the instrument documented changes in self-efficacy that were closely related to the treatment goals.

One of the more widely used measures of self-efficacy for coping strategies with cancer is the Cancer Behavior Inventory (CBI) [9]. The goal of the authors was to develop a comprehensive measure of self-efficacy strategies for coping with cancer that cover the major issues that confront cancer patients. There are two versions of the long form of the CBI, the original published in 1997 and a revision published in 2001 [6]. In addition, a brief version was published in 2011 [10].

The first version of the Cancer Behavior Inventory was published in 1997 [9]. Initially, 78 items were constructed based on interviews with persons with cancer and family members who had a relative with cancer, a review of the literature on coping with cancer (e.g., Weisman, 1984), input from health care professionals (e.g., radiation technologists, oncology nurses, and physicians), and the authors’ professional experiences counseling people with cancer. The list of 78 was reduced to 65 by eliminating redundant items and rewording others to make them more inclusive. Those items were formatted into a questionnaire in which persons with cancer were asked to rate, on a 9-point scale, the importance of each behavior and the difficulty in performing the behavior. A pilot testing on thirty-three persons with cancer was conducted to select items that were moderate on difficulty and high on importance. The final version of CBI consisted of 51 items randomly ordered with a 9-point likert scale ranging from “not at all confident” to “totally confident” in reference to the ability of the cancer patient to perform the behavior in the near future. The factor structure of the 51-item CBI was evaluated using principal components extraction and varimax rotation in a sample of 502 persons with cancer (see Merluzzi et al 1997 for specifics about the sample). Based on the factor analysis, eight items were eliminated that did not conform to simple structure; that is, they had roughly equal or low loadings or both on several factors or had one high factor loading of less than .45. Thus, the final version of the CBI contained 43 items. A six-factor solution emerged accounting for 53% of the item's variance. The six factors, internal consistencies, and brief descriptions are as follows: (a) Maintenance of Activity and Independence (a = .89) focuses on maintaining activity in spite of the disease and its treatments; (b) Coping With Treatment-Related Side Effects (a = .88) involves coping with the most dreaded aspects of the treatments, nausea, physical changes (e.g., hair loss) and limitations (e.g., lack of energy); (c) Accepting Cancer/Maintaining Positive Attitude (a = .87) reflects the dual tasks of maintaining a hopeful, positive state of mind while accepting the reality of the disease; (d) Seeking and Understanding Medical Information (a = .88) is concerned with personal involvement and active participation in the treatment of the disease; (e) Affective Regulation (a = .75) is an interesting combination of items that reflect, on the one hand, the expression of strong negative feelings and, on the other hand, denial, escape, and ignoring; and (f) Seeking Support (a = 77) reflects the initiation of support rather than the reception of support. The CBI was reliable in that internal consistency (Cronbach's alpha), for the entire scale was .96.

In 2001 Merluzzi, Nairn, Hegde, Sanchez, and Dunn proposed a second and shorter version of CBI consisting of 33-items [6]. The scale was completed by 280 participants and subjected to a factor analysis that was computed using a principal factors extraction and varimax rotation. A seven-factor solution was chosen to confirm the original six factors plus a new stress management scale as the seventh factor. The Kaiser-Meyer-Olkin (KMO) measure of sampling adequacy for the analysis was 0.86, which indicated an acceptable level of sampling adequacy. The seven-factor solution appeared to be optimal based on the variance accounted for (63.4%) and eigenvalues greater than one, except for the seventh factor that had an eigenvalue of 0.98.

Consistent with the original version, the seven factors were labeled as follows: (1) Maintenance of Activity and Independence (a= 0.86), (2) Seeking and Understanding Medical Information (a=0.88), (3) Stress Management (a=0.86), (4) Coping with Treatment-Related Side Effects (a=0.82), (5) Accepting Cancer/Maintaining Positive Attitude (a=0.86), (6) Affective Regulation (a= 0.81), and (7) Seeking Support (a= 0.80). The new factor (stress management) reflects the ability to cope with anxiety accompanying medical appointments and treatments. The internal consistency for the entire 33-item version was 0.94, which was only slightly lower than the 0.96 for the 43-item version.

More recently, Heitzmann, Merluzzi, Jean-Pierre, Roscoe, Kirsh, and Passik [10] developed a brief version of the Cancer Behavior Inventory Brief version (CBI-B) based on the need for a reliable, valid, easy-to- administer instrument, which was not burdensome for patients. The CBI-B was more feasible in clinical trials as a single-score measure of self-efficacy for coping that also had excellent psychometric qualities. The CBI-B was constructed by including two items with high-factor loadings from each factor of the first version of the CBI-L and two additional items from the stress management scale that was added to the second version (CBI 2.0). The final version of the scale was based on an EFA on one sample of cancer patients (N=735) and CFA in two other samples (N=370 & N=199) and consisted of 12 items. The internal consistency of the of the 12-item CBI- B, as indicated by the Cronbach a coefficient, was 0.84, 0.84, and, 0.88 for three different samples, respectively. The correlation of the CBI-B and the CBI-L (without corresponding CBI-B items in the CBI-L) was 0.95. The four factors were labeled: Independence and Maintaining a Positive Attitude, Participation in Medical Care, Coping and Stress Management, and Management of Affect. Table 1 contains information about each of the versions of the CBI.

**Table 1:** Descriptions of versions of the cancer behavior inventory

Name of the Scale	Authors, year	Number of items	Number of factors
CBI	Merluzzi et al., 1997	43	6
CBI 2.0	Merluzzi et al., 2001	33	7
CBI B	Heitzmann et al 2011	12	4

The purpose of the present meta-analysis, as noted earlier, is to focus more deeply on one of the more widely-used and well-developed measures of self-efficacy for coping, the Cancer Behavior Inventory, in order to provide more definitive analyses of that instrument. This close scrutiny of a measure can be used to evaluate the incremental validity of the measure as well as evaluate its utility in oncology settings. To date there have been syntheses of data supporting the value of social cognitive components in interventions for cancer patients [7]. Thus, the aim of the present work is to conduct stringent reviews of high quality measures to determine the exact relationship between self-efficacy for coping with cancer and psychosocial outcomes (i.e. distress and quality of life) at the meta-analytic level. This approach will test, on a single measure, the replicability of findings that were established across a variety of outcome measures that assessed distress or quality of life. In addition, this type of analysis is very relevant and timely given the increase number of studies showing that self-efficacy for coping with cancer is a critical factor in patients’ outcomes [11–14].

The following general questions provide a framework for this research: 1. Is self-efficacy for coping with cancer related to distress and quality of life (QoL). 2. Do self-efficacy for coping with cancer and the target psychological outcomes (i.e., distress and QoL) change in longitudinal studies, with or without intervention?

Corollary hypotheses are as follows:

Higher self-efficacy for coping with cancer, as measured by the Cancer Behavior Inventory, will be associated with better psychological adjustment and better QoL.

Interventions for cancer patients will improve self-efficacy for coping with cancer and have an impact on its related variables, improving distress and QoL of the patients.

## RESULTS

### Data sample

One-hundred eighty studies cited the different versions of the CBI scales (77 citation for the first version, 83 for CBI 2.0, and 20 of the brief version). Of the total 180 citations, only 47 studies used the scale. The studies in the sample of the 47 were categorized as follows: 1. Studies showing a correlation between self-efficacy for coping with cancer and QoL or psychological distress, (N=34), and 2. longitudinal studies - both observation and intervention studies (N=13). Of the whole sample of the 47 studies, 19 (15 cross sectional and 4 longitudinal) showed the information needed for this meta-analysis or the authors provided the information upon request [4, 6, 9, 11, 12, 14–27] and were used for the meta-analysis. Where the publication did not contain adequate information and authors did not provided requested data, the study was not included. As noted earlier, studies using only some factors of the scale were also excluded from the analysis. Finally, eight doctoral dissertations were found that used the CBI; 2 of these 8 studies correlated the CBI with variables measuring QoL or distress. Table 2 contains a summary of the data extraction for those studies used for the meta-analysis.

**Table 2:** Summary data including effect size for all correlational, cross-sectional studies with distress as the outcome

Autors and year	ES	LL	UL	Var	SE	W	Res.	R-Sig.	N
Abby N, Diehl, 2014	−1,303	−1,779	−0,828	0,059	0,242	4,773	−0,351	0,726	100,000
Albrech et al, 2013	−1,094	−1,435	−0,754	0,030	0,174	6,005	0,244	0,807	175,000
Chirico et al, 2015	−1,250	−1,660	−0,840	0,044	0,209	5,348	−0,216	0,829	130,000
Heitzmann et al, 2011b	−1,218	−1,545	−0,890	0,028	0,167	6,129	−0,129	0,897	199,000
Heitzmann et al, 2011c	−1,317	−1,562	−1,072	0,016	0,125	6,917	−0,466	0,642	370,000
Henselmans et al, 2010	−1,317	−1,815	−0,820	0,064	0,254	4,586	−0,380	0,704	92,000
Howspiean et al, 2010	−1,552	−1,942	−1,162	0,040	0,199	5,537	−1,150	0,250	165,000
Li et al, 2015	−0,873	−1,326	−0,420	0,054	0,231	4,960	0,836	0,403	92,000
Mazenac et al, 2012	−1,469	−2,023	−0,915	0,080	0,283	4,142	−0,752	0,452	80,000
McGinty et al, 2016	−1,250	−1,623	−0,878	0,036	0,190	5,701	−0,222	0,824	157,000
Merluzzi et al, 1997	−0,860	−1,025	−0,695	0,007	0,084	7,593	1,085	0,278	672,000
Mosher et al, 2010	−1,008	−1,487	−0,529	0,060	0,244	4,741	0,450	0,652	87,000
Nairn et al, 2004	−1,666	−2,122	−1,210	0,054	0,233	4,935	−1,418	0,156	128,000
Passik et al, 2002	−0,994	−1,306	−0,682	0,025	0,159	6,283	0,557	0,577	200,000
Philip et al, 2013	−1,580	−2,035	−1,126	0,054	0,232	4,953	−1,159	0,247	124,000
Shino group a 2010	−0,408	−0,808	−0,008	0,042	0,204	5,442	2,397	0,017	103,000
Shino group b 2010	−1,711	−2,178	−1,244	0,057	0,238	4,842	−1,540	0,124	125,000
Zachariae et al, 2002	−0,711	−0,934	−0,488	0,013	0,114	7,113	1,657	0,097	350,000
**Overall (random-effects model)**									
	**k**	**ES**	**LL**	**UL**	**Sig**.	**Var**	**SE**	**N**	
	18	−1.17	−1.33	−1.01	0.00	0.01	0.08	3349	

ES= effect size; LL= lower limit; UL= upper limit; Var= Variance; W= Weight; Res.= standardized residuals R-Sig.= Statistical significance of standardized residual; Sig.= Statistical Significance; k= total number of studies; SE= Standard Error.

### Correlation of the CBI with distress and quality of life: cross sectional meta-analysis

Of the 47 studies identified, 14 journal article [4, 9, 11, 14–18, 20–23, 27, 28] used a version of the CBI and a measure of distress. To these two dissertation thesis were added [29, 30], thus, a total of 16 studies were analyzed. In those studies coping self-efficacy was moderately to strongly associated with depression and anxiety dimensions according to Cohen's criteria [31]. However, overall random effect size of the relationship between CBI and distress measures was -1.17 (Table 2, Figure 2), which would be considered a very large effect size.

**Figure 1:**
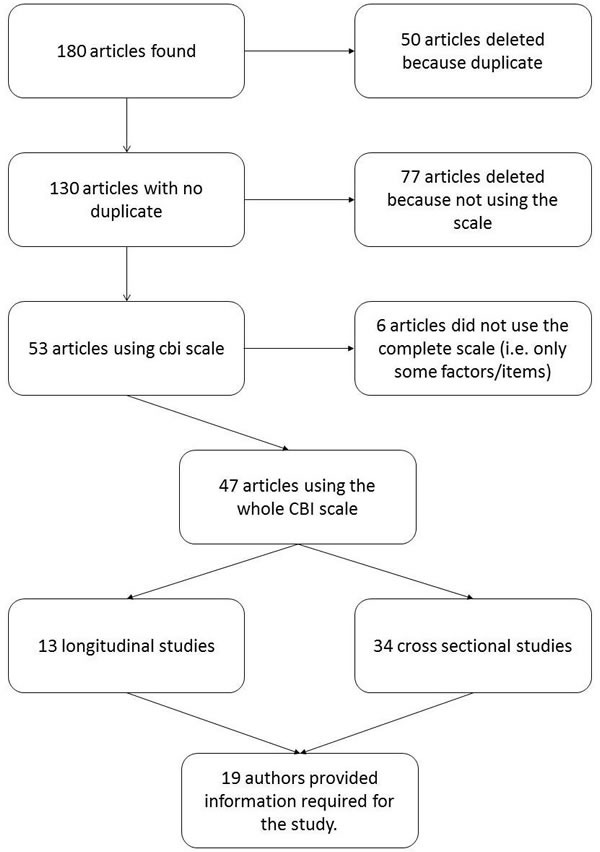
Flow chart of inclusion and exclusion of studies

**Figure 2:**
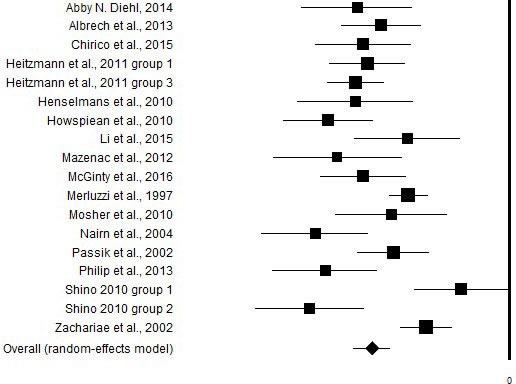
Graphic representation of the effect size of the correlational between the CBI and distress

**Figure 3:**
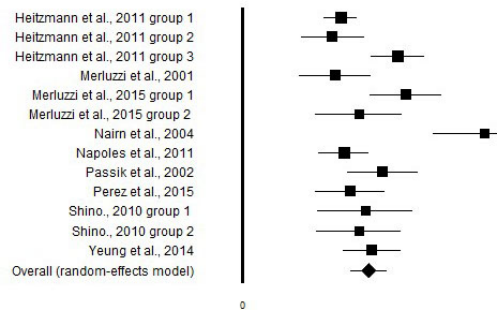
Graphic representation of the effect size of the correlational between the CBI and quality of life

Eight studies evaluated the relationship between CBI and QoL [6, 12, 21, 24, 26–28, 32], three of those [21, 27, 28] included distress and were reported with the distress outcomes too in Table 3. One dissertation was also included in this analysis [29]. The overall effect size across the studies, ES=1.22, showed a positive relationship between the CBI and QoL outcomes, and would be considered a very large effect size according Cohen's criteria [31] (Table 3, Figure 3).

**Table 3:** Summary data including effect size for all correlational, cross-sectional studies with quality of life as the outcome

Author, year	ES	LL	UL	Var	SE	W (%)	Res.	R-Sig.	N
Heitzmann et al, 2011 group a	0,953	0,792	1,113	0,007	0,082	9,808	−0,953	0,341	735
Heitzmann et al, 2011 group b	0,873	0,567	1,178	0,024	0,156	8,083	−1,137	0,255	199
Heitzmann et al, 2011 group c	1,500	1,244	1,756	0,017	0,130	8,716	0,994	0,320	370
Merluzzi et al, 2001	0,899	0,555	1,243	0,031	0,176	7,588	−1,012	0,311	159
Merluzzi et al, 2015 group a	1,580	1,236	1,924	0,031	0,175	7,590	1,183	0,237	214
Merluzzi et al, 2015 group b	1,124	0,710	1,538	0,045	0,211	6,719	−0,284	0,776	121
Nairn et al, 2004	2,339	1,841	2,836	0,064	0,254	5,770	3,691	2.237.916.277.492,140	150
Napoles et al, 2011	0,980	0,739	1,221	0,015	0,123	8,895	−0,804	0,421	330
Passik et al, 2002	1,355	1,018	1,693	0,030	0,172	7,674	0,420	0,675	200
Perez et al, 2015	1,043	0,707	1,379	0,029	0,172	7,690	−0,555	0,579	176
Shino, 2010 group a	1,186	0,728	1,644	0,055	0,234	6,204	−0,100	0,920	102
**Overall (random-effects model)**									
**k**	**ES**	**LL**	**UL**	**Sig**.	**Var**	**SE**	**N**		
13	1,220	1,044	1,39	0.0	0,008	0,089	3162		

ES= effect size; LL= lower limit; UL= upper limit; Var= Variance; W= Weight; Res.= standardized residuals R-Sig.= Statistical significance of standardized residual; Sig.= Statistical Significance; k= total number of studies; SE= Standard Error

An ANOVA was performed between the dissertation theses and the journal articles in order to determine if there was a significant difference in the results that might be indicative of an publication bias effect. Results of the ANOVA showed no significant effect size differences between the two groups. (Sig.= 0.65)

### Systematic evaluation of longitudinal studies

Fourteen studies evaluated self-efficacy for coping with cancer in longitudinal designs. Because the focus of this meta-analysis was on the relationship of the CBI with QoL and distress, longitudinal studies were included in the cross-sectional analysis if those relationships were reported or could be determined. However, some studies did not include information about correlation of the CBI with either QoL or distress during any of the time points of the study and, therefore, those studies were evaluated in terms of the change in the CBI over time in observational studies or due to an intervention. Some studies did not investigate any psychological outcomes in relationship with self-efficacy for coping with cancer. In other studies self-efficacy for coping with cancer was investigated as moderator or mediator in relationship with other outcome variables (no QoL or distress evaluated in any time points of the study).

The sample of longitudinal studies can be differentiated between intervention [33–39] (n=7) and observational studies [11, 13, 15, 18, 23, 40–42] (n=8). The intervention studies did not necessarily target self-efficacy for coping with cancer as a component in the treatment, but merely evaluated changes in self-efficacy as a variable in the studies. Five of 7 studies found an improvement in self-efficacy for coping in the treatment compared to the control group [33, 34, 37, 38, 43]; one study did not find any differences [39], one found the self-efficacy for coping with cancer deteriorated in control group, whereas the intervention group did not show any changes, which could not be considered as a feasible effect of the intervention [36].

Eight studies evaluated self-efficacy for coping with cancer using longitudinal observational designs. Three of these found that the CBI predicted future stress or QoL outcomes [11, 13, 40]. Three studies did not find any correlation/predictor effect of coping efficacy with cancer [15, 18, 42], one found an improvement of the variable after a medical consult (mammogram) [41], one did not show/provided any information [23].

## DISCUSSION

Generally, the meta-analysis showed that the relationship between self-efficacy for coping and important outcomes, distress and QoL, were consistent with theory [44].

The results indicated that perceived self-efficacy, a belief in one's ability to execute coping strategies in confronting cancer, was very strongly negatively associated with distress outcomes. (ES d=-1.17). These results are consistent with findings in other areas of health psychology, for example in a recent meta-analysis by Mathews involving 5315 youth, there was a large significant overall effect size of r = −0.36 (d=-0.61) for the association between emotional self-efficacy and anxiety [45]. Youth with higher anxiety levels perceive themselves as less efficacious in their ability to activate or inhibit emotional responses.

All the studies included in this meta-analysis showed significant and positive correlations between self-efficacy for coping with cancer, evaluated with CBI, and quality of life as well. The overall results showed a positive effect size of 1.24. Thus, as noted in Merluzzi et al 2001: “because higher levels of efficacy are characterized by a sense of agency or control, a highly efficacious cancer patient may perceive some causal relationship between coping behaviors executed and certain desired outcomes, such as level or type of quality of life” [6, 46].

The strong relationship of self-efficacy and outcomes in the current study could be the result of the specificity of the scale (CBI), which targets specific coping strategies that are closely aligned with positive outcomes in adjusting to cancer. However, the results are consistent with the general theory, which states that compared to those with low efficacy, highly efficacious people demonstrate less anxiety and better adjustment in stressful situations and consistent with prior results in which self-efficacy is positively related to QoL [6].

From a theoretical point of view, self-efficacy for coping with cancer could be considered as a mutable skill that can be taught in the context of an intervention that focus on enhancing the patient's sense of agency in coping with cancer. Then self-efficacy may be a mediator between the sometimes overwhelming effect of diagnosis and treatment and Qol or distress outcomes. Thus, acquiring self-efficacy or the confidence to cope effectively may be transformational in terms of mitigating the negative effects of cancer diagnoses and treatments.

The review of longitudinal studies did not clarify the role of self-efficacy for coping with cancer in revealing the relationship of the CBI with the key variables focused in this meta-analysis, in a meta analytical way, this due to the paucity of information provided from authors of the studies when contacted. However from a narrative review of those studies it seems clear that intervention in cancer patients may help improving patients’ self-efficacy, found as result in five of the seven studies evaluated

Studies of other health conditions, for example in asthma or in chronic disease, showed how specific interventions focused on self-efficacy resulted in having an important long-term impact on distress and QoL outcomes.[47–49] For that reason it is very important to understand the role of self-efficacy for coping with cancer in longitudinal, RCT experimental design, with representative sample of subjects, in order to establish and confirm the changes in the CBI and its relation or impact on other important outcomes. Furthermore, evidence for the effects of interventions for cancer patients on self-efficacy expectations across a number of domains and measures are in a meta-analysis study focusing on Randomized Controlled Trials (RCT) [manuscript in preparation] The result of this meta-analysis that included a treatment and control condition as well as an assessment of a self-efficacy construct, indicated that across all studies there was an effect size between small and medium (g=0.338). Moreover, there was a moderating effect of treatment format such that effects for group-based interventions (g=0.702) were statistically distinguishable from effects for individual-based (g=0.218) interventions (P=.015). The superiority of the group format was consistent with self-efficacy theory in that the group format may enhance self-efficacy through processes that are very central to Social Cognitive Theory - social modeling, persuasion in a group context, and support. In a systematic review of the literature on interventions for cancer patients, it does appear that interventions that have a social cognitive focus are more effective in producing behavior change [7]. Thus, whereas there is general information about the impact of interventions on self-efficacy, the focus on specific interventions designed with social cognitive components that impact efficacy and the use of psychometrically sound measures like the CBI would help to establish a more firm empirical base for interventions for cancer patients that are theoretically based.

For example, a study by Pellino et al. (1998)[50] embodies these recommendations. Those authors have been focused their attention on a empowerment interventions in order to increase self-efficacy for coping in orthopedic patients. Similar studies would advance research in coping with cancer and lead to more substantive meta-analyses.

These recommendations are also relevant in that there is a new emphasis on empowering cancer patients during all phases of the cancer care trajectory. This new thrust puts self-efficacy generally at the forefront, and coping efficacy as one aspect of empowerment. A recent review of literature [51], after analyzing different studies that included self-efficacy and self-management interventions, developed the Stanford Chronic Disease Self-Management Program (CDSMP). The program is currently used in all US states and has been adapted for use in 25 countries worldwide. Since 2010, the program has reached more than 150,000 people in the US alone. CDSMP has a large evidence base and has been described in detail in previous publications [51]. Briefly, CDSMP is based on social cognitive theory and specifically self-efficacy theory and is designed to enhance personal efficacy (i.e., confidence in one's ability to manage different aspects of one's health functioning) through skills mastery, reinterpretation of symptoms, modeling, and social persuasion. Results of a typical CDSMP study [52] showed significant improvements in cancer patients in most of the psycho-social variables took, even if no specific measure of self-efficacy for coping with cancer are mentioned. As claimed by the authors “more attention is needed to the development of a common language for measuring outcomes of self-management interventions among cancer survivors” [51].

Limitations of the work using the CBI to date include a predominance of cross sectional studies over longitudinal studies. Cross sectional studies are easier to perform, but longitudinal RCTs are more valuable in terms of documenting change in self-efficacy and demonstrating its relationship to change in other outcomes such as distress and QoL. Attempts were made to contact all the authors of studies that utilized measures of coping efficacy for cancer, yet failed to report correlational information. The non-response of some of these individuals undoubtedly limited the scope and power of this study. Another limitation is that the researchers selected only those studies written in English. The reader should be aware that because of this limitation, the sample of included studies might be biased. However, according to Thornton and Lee (2000) [53], all meta-analyses that do not review all studies are similarly biased. Still, a retrospective review of 303 meta-analyses using the English language restriction found no systematic bias [54]. Therefore, despite this limitation, some confidence is warranted when evaluating this study's outcomes.

Meta-analyses are unique in that they offer a systematic investigation of the literature within field. While this aspect of a meta-analysis may be its strength, it is also its weakness. Due to the collection procedures in meta-analyses, studies that remain unpublished are ultimately left out. Failing to include these studies in the analyses biases the results in favor of those that were fortunate enough to be published [55, 56]. The studies that fail to be published are stored away and typically never see the light of day.

In order to account for this file drawer problem, a fail safe N was calculated. The fail safe N is a calculation of the number of studies with little or no effect size that would be needed to be included in the analysis to reduce the overall correlation effect size to a non-significant level. Considering studies with a small, but non-zero correlation effect size, it would require 2011 studies to lower the estimated correlation to a level that is no longer significant, the value is above the Rosenthal's rule of thumb (5k+10 = 65).

The review of longitudinal studies did not clarify the role of self-efficacy for coping with cancer in revealing the relationship of the CBI with the key variables focused in a meta-analytic way. This it is due to 1. the exiguity of information provided in the studies or from the authors, that didn't allow to use these in a meta-analytic approach; and 2. the lack of homogeneity of studies in that each used different interventions, different outcome variables, and almost did not focus on self-efficacy in the intervention. These limitations does not allow us to clarify which are the specific causal relationship between these variables. According to other studies in other health related contexts, specific intervention on self-efficacy would be able to cause a reduction of the stress.[57] Future researches are solicited to carefully evaluate this relationship to confirm our hypotheses.

## MATERIALS AND METHODS

This meta-analysis focused on data published in peer-reviewed journal articles that have reported the administration of the CBI (any of the three versions) to participants, and have been published between 1997 and July 2016. This systematic review has been conducted in accordance with a defined set of criteria established in the Preferred Reporting Items for Systematic Reviews and Meta-Analyses (PRISMA) guidelines [58].

### Search strategy

A comprehensive search of the electronic database Scopus was conducted to identify articles citing the original publications on the development or revision of the CBI scales. Those publications that referenced the original publications were then searched using the search terms ‘Cancer Behavior Inventory’; ‘CBI’; ‘CBI-B’.

### Inclusion and exclusion criteria

The inclusion criteria were papers in all languages that have reported administering the CBI or a modified version of the CBI, and were published in peer-reviewed journals or published dissertation thesis. Exclusion criteria were papers not administering the CBI, administering only few factors of the scale, duplicate papers, commentaries, book chapters, literature reviews, clinical guidelines, conference proceedings or abstracts and study protocols.

### Review strategy and procedure

The initial search identified 180 publications in scientific peer reviewed journals. Once duplicates were removed, 130 publications were included in the preliminary set. The titles and abstracts of the publications were inspected using the inclusion criteria and 77 were excluded. After full-article review one more study was excluded, leaving 47 papers in the analysis. A collection of eight doctoral dissertation theses was found on internet based on google search, and were included in the meta-analysis.

A detailed procedure for extracting relevant information from the journal papers included: the name of the paper; author(s); year of publication; number of participants; participant mean ages; study design; text that referred to the results relating to the CBI, as well as r scores, and p levels for significant effects (p) involving the variables of interest (CBI, distress, QoL). Figure 1 contains a flow chart describing the selection of studies.

### Data analysis

Random effects meta-analysis was conducted using the Prometa3 software (Internovi 2015). Where CBI scales were scored such that higher scores indicated higher self-efficacy for coping with cancer, the sign of the correlation coefficient was changed to ensure consistency with measures of stress (i.e., measures of distress or adjustment were reversed in order to have uniform interpretation of the results) and QoL. In cases were studies reported multiple effect sizes (e.g.,multiple rs), they were examined to determine whether the effect sizes were independent of one another before utilizing both. For example, a study that utilized three different cancer patient samples from different locations and reported three different correlation coefficients between self-efficacy and distress would be considered independent and were coded as three different effect sizes. Pooling was undertaken based on the Fisher's z transformation of the correlation coefficient, where the standard error was calculated as $1/\sqrt{n-3}$ [59]. Back transformed correlations are displayed. Where there were fewer than three samples reporting correlation coefficients no pooling of the correlations was undertaken.

## Abbreviations

CBI: Cancer Behavior Inventory
CBI-B: Cancer Behavior Inventory-Brief form
CBI-L: Cancer Behavior Inventory Long form
QoL: Quality of Life
CDSMP: Chronic Disease Self-Management Program
RCT: Randomized Controlled Trial
KMO: Kaiser-Meyer-Olkin
ANOVA: Analysis of Varianca
